# LPS-Stimulated Human Skin-Derived Stem Cells Enhance Neo-Vascularization during Dermal Regeneration

**DOI:** 10.1371/journal.pone.0142907

**Published:** 2015-11-13

**Authors:** Tobias Kisch, Caroline Weber, Daniel H. Rapoport, Charli Kruse, Sandra Schumann, Felix H. Stang, Frank Siemers, Anna E. Matthießen

**Affiliations:** 1 Department of Plastic Surgery and Hand Surgery, University of Lübeck, Lübeck, Germany; 2 Fraunhofer Research Institution for Marine Biotechnology, Lübeck, Germany; 3 Department of Plastic Surgery and Hand Surgery, BG-Kliniken Bergmannstrost, Halle, Germany; Wake Forest Institute for Regenerative Medicine, UNITED STATES

## Abstract

High numbers of adult stem cells are still required to improve the formation of new vessels in scaffolds to accelerate dermal regeneration. Recent data indicate a benefit for vascularization capacity by stimulating stem cells with lipopolysaccharide (LPS). In this study, stem cells derived from human skin (SDSC) were activated with LPS and seeded in a commercially available dermal substitute to examine vascularization *in vivo*. Besides, *in vitro* assays were performed to evaluate angiogenic factor release and tube formation ability. Results showed that LPS-activated SDSC significantly enhanced vascularization of the scaffolds, compared to unstimulated stem cells *in vivo*. Further, *in vitro* assays confirmed higher secretion rates of proangiogenic as well as proinflammatoric factors in the presence of LPS-activated SDSC. Our results suggest that combining activated stem cells and a dermal substitute is a promising option to enhance vascularization in scaffold-mediated dermal regeneration.

## Introduction

The outer barrier of the human body skin has various important functions such as protection from pathogens and thermoregulation. Hence, full-thickness skin damage resulting from indispensable debridement of deep burn injuries or chronic wounds indicates severe physiological problems for the patient. Although split-skin grafting remains the “gold standard” for treating damaged areas due to its easy and fast harvesting, availability is limited [1]. Moreover, restoring the full barrier function and mobility of the skin will not occur unless dermal and epidermal layers are completely rebuilt [2]. Additionally, solely split-skin grafting is insufficient for a good functional and esthetical outcome.

Therefore, skin tissue engineering has emerged as an alternative therapeutic option. In this context, three-dimensional biodegradable scaffolds are serving as a backbone for infiltrating cells and new vessel formation. Besides cadaver donor skin, certified dermal replacement materials are used in different clinical settings [3]. However, the time intensive vessel growth, epithelial restoration and the risk of scaffold infection remain serious problems. Thus, enhancing vascularization is a challenge of scaffold-mediated tissue engineering.

In order to enhance vascularization in wounded areas, recent approaches fostered the activation of scaffolds by the use of recombinant molecules or DNA vectors to induce a temporary release of proangiogenic factors [4]. Besides, genetically modified cells or stem cells have been used in clinical and preclinical trials [5–8].

Previously, we have shown that human stem cells derived from skin and sweat glands do not only have a multipotent differentiation capability *in vitro*, but do also improve vascularization during skin regeneration *in vivo* [9–12]. Other glandular stem cell populations of murine origin have also been shown to accelerate wound healing in the context of scaffold-based dermal regeneration [13,14]. The required number of cells needed in a clinical setting is still huge.

Mostly, stem cells mediate their beneficial effects by complex paracrine actions [11]. This led us to the assumption that enhanced secretion of these signals by stem cells would result in fewer cells required for treatment.

One way to activate stem cells is to stimulate them with endotoxins such as lipopolysaccharide (LPS). For example, human mesenchymal stem cells (hMSC) are known to increase the secretion of factors that are playing a major role in angiogenesis and recruitment of progenitor cells in wounded tissue, such as vascular endothelial growth factor (VEGF), fibroblast growth factor 2 (FGF-2) and insulin-like growth factor 1 (IGF-1) [15].

The purpose of this study was to enhance cytokine and growth factor secretion by skin-derived stem cells seeded in a scaffold. Additionally, we aimed to decrease the number of cells, that is needed to obtain comparable results in dermal angiogenesis with respect to previous studies. Therefore, stem cells were isolated from human full skin biopsies and characterized by the expression of nestin, a marker for adult stem and progenitor cells [10,16]. We stimulated the cells with LPS *in vitro* and analyzed gene and protein expression of proangiogenic factors. Furthermore, we examined the vascularization potential of LPS-stimulated cells in an *in vivo* full-thickness skin defect model.

## Materials and Methods

### Ethics Statement

All experiments were performed according to Helsinki guidelines, in compliance with national regulations for the experimental use of human material. Utilization of human biopsies for research purposes was approved by the Institutional Ethics Committee at the University of Lübeck. All patients gave written informed consent.

The experimental procedures with animals were approved by the Ministry of Energy, Agriculture, the Environment and Rural Areas (MELUR) and were conducted in accordance with the German legislation on protection of animals and the National Institutes of Health Guide for the Care and Use of Laboratory Animals (Institute of Laboratory Animal Resources, National Research Council).

### Cell isolation, culture and seeding

Skin-derived stem cells (SDSC) were isolated from human full skin biopsies as it was reported earlier [17,18]. First, skin tissue (2 cm^3^) was freed of adhering fat tissue and chopped into small pieces with surgical scissors. Shortly after, the tissue was treated twice (20 and 15 min) with digestion medium containing HEPES-Eagle medium (pH 7.4), 10 mM HEPES buffer, 70% (v/v) modified Eagle´s medium, 0.5% (v/v) Trasylol (Bayer AG, Germany), 1% (w/v) bovine serum albumin, 2.4 mM CaCl_2_ and collagenase (0.63 PZ/mg, Serva, Germany) at 37°C. Prior to each digestion, the preparation mixture was aerated with a mixture of oxygen and carbon dioxide (95% (v/v)). In between the two digestion steps, the remaining tissue pieces were washed and mechanically minced with small surgical scissors again. After the second digestion step, the remaining tissue fragments were dissociated into even smaller pieces by up-and-down suction through different glass pipettes with progressively more restrictive orifices (10, 5 and 2 ml pipettes) and filtered through a nylon mesh (250 μm mesh). After centrifugation (850 rpm, 5 min) and further purification by washing in Dulbecco’s modified Eagle’s medium (DMEM, Gibco, Germany) supplemented with 20% fetal bovine serum (FBS), 1 U/ml penicillin and 10 mg/ml streptomycin (all PAA Laboratories, Austria), the cells were seeded onto 25 cm^2^ flasks. Further cultivation was carried out in 75 cm^2^ culture flasks in DMEM supplemented with 10% FBS (10% FBS-DMEM).

This simple technique for isolating and propagating stem cells from human skin led to a crude mixture of skin-derived stem cells characterized by the expression of nestin in 20% of the cells [17].

Stem cell cultivation in 3D was performed using Integra matrix (IM; Integra LifeScience Corporation, NJ, USA), a scaffold based on bovine collagen fibres cross-linked with glucosaminoglycans, which form a porous biodegradable structure. As a temporal epidermis, the collagen structure is covered with a silicone layer. For the 3D cultivation, the IM was carefully washed in phosphate buffered saline (PBS), 6 mm pieces in diameter were punched and placed in 12-well plates with the silicone layer pointing downwards. The liquid in the IM was reduced by using filter paper and 3 x 10^5^ stem cells (SC) were diluted in 30 μl 10% FBS-DMEM and transferred to the scaffold. Before adding 1 ml 10% FBS-DMEM, the SC seeded scaffolds were further incubated for 1 h at 37°C to improve the attachment of the SC to the matrix.

### LPS-stimulation

Lipopolysaccharid (LPS) was obtained from E.coli 11:B4 (Sigma Aldrich). For SC stimulation in 2D cultures LPS was diluted in the proper concentration (10 ng/ml and 100 ng/ml) in 10% FBS-DMEM and directly added to the cells. LPS-stimulation in 3D was performed by removing the medium, adding 30 μl of 10 ng/ml LPS and incubating for 2 h at 37°C. Subsequently, the scaffolds were washed once with PBS and further incubated in 10% FBS-DMEM.

### Immunocytochemistry

For immunocytochemical staining 1 x 10^5^ SDSC were seeded on 4-well chamber slides each. The next day, stem cells were stimulated with 100 ng/ml LPS for 1 h at 37°C, washed in PBS, fixed with 4% paraformaldehyd for 10 min at room temperature (RT) and rinsed three times with PBS. Permeabilization was performed by using 0,1% Triton X in PBS (10 min, RT). After washing carefully with PBS, a blocking step with 10% normal goat serum (Vector Laboratories, CA, USA) diluted in PBS was performed for 20 min at RT to saturate non-specific binding sites. Next, SDSC were incubated with a specific primary antibody anti TLR-4 (1:100, mouse, abcam, UK) and anti LPS (1:100, mouse, abcam, UK) diluted in TBST containing 0.1% bovine serum albumin (PAA Laboratories, Austria) in a humid chamber for 1 h at 37°C. Before adding the secondary antibody, the SDSC were carefully rinsed with PBS. Cells were incubated with Cy3-labeled anti mouse IgG (1:400, Dianova, Germany) or FITC-labelled anti-mouse IgG (1:200, Dianova, Germany) in a humid chamber for another 1 h at 37°C. The slides were washed again three times with PBS, covered with Vectashield mounting medium (Vector Laboratories, CA, USA) and analyzed with a fluorescence microscope (Observer, Zeiss, Germany).

### RNA isolation and reverse-transcriptase-PCR

After 4 h, 24 h and 48 h of LPS-stimulation, RNA was obtained from trypsinized and centrifuged SDSC and from the SDSC-seeded scaffolds respectively. As a control unstimulated SC and unstimulated SC-seeded scaffolds were used. Prior to RNA isolation, the scaffolds were shock frozen in liquid nitrogen and pulverized with a mortar and pestle to simplify the isolation procedure. Total RNA isolation was performed using the RNeasy Plus Mini kit and the QIAcube for automated RNA isolation according to manufacturer´s protocols (both Qiagen, Germany). RNA concentration was determined by measuring the absorbance at 260 nm in a nanodrop spectrophotometer. cDNA was synthesized from 1 μg total RNA using the QuantiTect reverse transcription kit including a gDNA digestion step. qPCR was carried out with 1 μl cDNA in a 25 μl reaction volume using the QuantiFast SYBR Green PCR kit and commercially available ready-to-use QuantiTect Primers: TLR 4 (QT01670123) and GAPDH (QT01192646; both Qiagen). Real-time quantifications were performed in duplicates for 45 cycles using the Mastercycler ep realplex (Eppendorf, Germany). The specificity of the amplified product was confirmed by melting point analysis. The fluorescence threshold value was calculated using the Mastercycler ep realplex 1.5 software and the CalQplex algorithm (Eppendorf, Germany). Gene expression levels were determined by applying the ΔΔCt method employing GAPDH as endogenous control.

### Cell visualization in a collagen scaffold

In order to observe the SDSC in their 3D environment, SDSC were seeded in Integra matrix and stained 7 days after seeding with Phalloidin. Therefore, the scaffolds containing cells were fixed in 4% paraformaldehyde for 1 h, pre-embedded in low-melt agarose and solidified at 4°C for 1 h in order to allow re-expansion of the tissue and proper mounting on the slides. Afterwards, the scaffolds were cut out of the agarose and frozen in Tissue Tek at -20°C overnight. 8 μm cryosections were made and washed in 40°C warm PBS to dilute the tissue tek and the agarose. Next, the sections were fixed again with 4% paraformaldehyde for 10 min and rinsed in PBS. After permeabilizing with 0.1% Triton X for 25 min, the sections were stained for 30 min at RT with TRITC-conjugated Phalloidin (1:200, Sigma-Aldrich) to detect the cytoskeleton. The nucleus was visualised through DAPI-staining (1:1000; diluted in PBS) for 5 min at RT. The sections were finally rinsed in PBS, mounted, and analyzed with a fluorescence microscope (Observer; Zeiss, Jena, Germany).

### Quantification of metabolic activity in the scaffold

SDSC (3 x 10^5^) were seeded in a 6-mm-diameter scaffold. At different time points, scaffolds were incubated for 1 hour in fresh medium containing 0.5 mg/ml of MTT (Sigma-Aldrich). Next, the medium was removed and replaced with 150 μl of DMSO (Sigma-Aldrich). To quantify metabolic activity, absorbance at 560 nm was measured in the DMSO containing soluble formazan blue. DMSO was used as blank.

### Angiogenesis array profile

Secretion of angiogenic factors was analyzed using a human angiogenesis protein array (R&D Systems, Minneapolis, USA). For this approach, supernatants of three unstimulated and three LPS-stimulated SDSC-seeded scaffolds (3 x 10^5^ stem cells/6 mm-scaffold cultured in 1 ml medium) were collected 24 h after LPS treatment and analyzed following the manufacturer’s instructions (pooled 1:3). 10% FBS-DMEM was used as negative control.

### Matrigel tube formation assay

150 μl of matrigel (BD bioscience) was added into each well of a 24-well plate. 2.5 x 10^4^ human umbilical vein endothelial cells (HUVEC; Promocell, Heidelberg, Germany) were seeded per well after 30 min incubation at 37°C. Four different media were analyzed on their effect on capillary-like structure formation: Endothelial Growth Medium (EGM; PromoCell, Germany) as positive control, 10% FBS-DMEM, and conditioned 10% FBS-DMEM obtained from 24 h unstimulated and LPS-stimulated SDSC-seeded scaffolds. For morphological analysis microscopic images were taken after 3 h of incubation. The experiment was repeated three times and the number of branching points and cumulative tube length were quantified in six random fields with Angiogenesis Analyzer implemented in ImageJ [19].

### Scratch assay

A scratch assay was performed using HUVEC. Cells were seeded in 24-well-plates and cultivated in EGM up to confluency. The scratch was performed using a 100 μl pipette tip. Scratched cells were removed by rinsing with PBS. Further cultivation took place under various conditions. EGM was used as a control. Furthermore, 10% FBS-DMEM, and conditioned 10% FBS-DMEM from SDSC-seeded scaffolds and LPS-stimulated SDSC-seeded scaffolds were used. After 8 h pictures were taken to document scratch reduction. Results were expressed as the percentage of the original gap surface covered with cells. The assay was performed three times.

### Scaffold-based dermal regeneration model

20 athymic nude mice (8 weeks old; Jackson Laboratories) were distributed in 2 groups à 10 animals. Before transplantation, animals were anesthetized with ketamine (10 mg/kg) and xylazin (2.4 mg/kg) via intraperitoneal injection. Under general anaesthesia, a full skin defect was created (8 mm diameter) in the back of the animals and the skin was replaced either by a SDSC-seeded scaffold (group 1) or a LPS-stimulated SDSC-seeded scaffold (group 2). Scaffolds were fixed to the wound by using non-absorbable sutures, and wounds were bandaged with a foil dressing (Tegaderm; Fresenius Kabi, Bad Homburg, Germany). One week after transplantation, animals were sacrificed by cervical fracture and exsanguination and the scaffolds including the surrounding skin were removed for further analysis.

### Quantification of vascularization levels

In order to quantify vascularization levels, tissue transillumination and digital segmentation were performed as described before [20]. After seven days, skin from the back of the animals was removed, quickly placed over a transilluminator (LP 5000K; Hama, Monheim, Germany) and documented with a digital camera (Olympus Camera C-5060, 5mpx). The pictures were processed for digital segmentation using VesSeg-Tool (http://www.isip.uni-luebeck.de/index.php?id=150). With the Hysteresis-threshold algorithm the vessel area was detected and the white-percentage representing the vessel area was calculated. With a second tool (skeletization) the vessel length was analyzed. Results were expressed as percentage of vascularization (vessel area and vessel length) compared to normal skin.

### Quantification of wound contraction

Harvested skin wounds were embedded in paraffin and then cut in 6 μm serial sections from the center of the wound. The slide presenting the largest wound diameter was defined as the wound center. The sections were stained with hematoxylin and eosin (H&E) and evaluated with a microscope (Axioskop 2 mot plus; Zeiss, Jena, Germany). Wound contraction was quantified by comparing the relative wound margin distance as described before [21].

### Statistical analysis

For statistical analysis the unpaired Student t-Test was used. Data was expressed as mean±SEM. Differences among means were considered significant when p<0.05.

## Results

### LPS uptake in skin-derived stem cells

**Fig 1:**
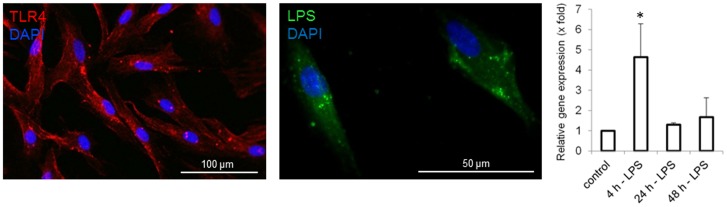
LPS Uptake. An immunocytochemical staining revealed the expression of TLR4 in skin-derived stem cells (SDSC). The incorporation of LPS could be shown by staining of LPS in SDSC. The LPS uptake led to a significantly enhanced expression of TLR4 mRNA 4 hours after stimulation with 10ng/ml LPS. Afterwards TLR4 expression returned to normal level. *p<0.05.

LPS is known to be recognized via the toll-like receptor 4 (TLR-4)/MD2 receptor complex and binding to the co-receptor CD14. An immunocytochemical staining revealed the expression of TLR-4 on the surface of human skin-derived stem cells (SDSC), after cells had been successfully isolated from human full skin biopsies. The addition of LPS, obtained from E.coli 11:B4, to the growth medium led to an incorporation of LPS into the cells, as confirmed by immunocytochemical staining. Subsequent intracellular uptake of LPS resulted in significantly enhanced expression of TLR-4 mRNA within 4 h. At later time points (24 h or 48 h after stimulation) TLR-4 mRNA expression had again decreased to baseline levels (Fig 1).

### Skin-derived stem cell growth on a collagen scaffold

**Fig 2:**
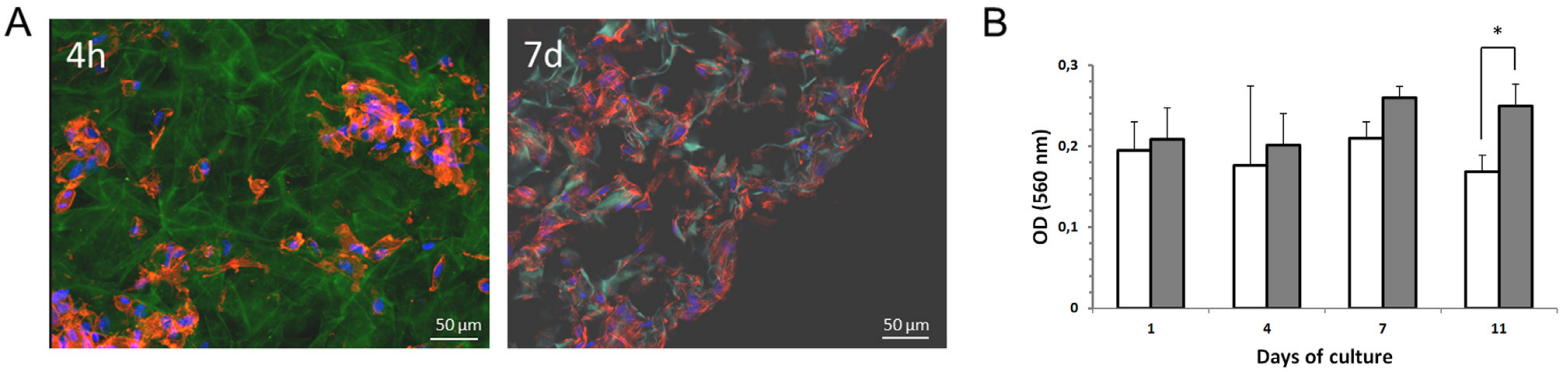
SDSC-seeded scaffolds. Cell matrix interactions in the scaffold (Autofluorescence/green) were evaluated 4 hours after cell seeding. Cells are presented in blue (DNA: 40,6-diamidino-2-phenylindole (DAPI)) and red (F-actin: phalloidin). After 7 days a homogeneous distribution of SDSC in the scaffold could be shown (A). Cell metabolic activity in the scaffold was evaluated for different time points by 3-(4,5-dimethyl-2-thiazolyl)-2,5-diphenyl-2H-tetrazolium bromide (MTT) assay. The metabolic activity remained significantly unchanged after 7 days of culture (white bars: LPS-stimulated SDSC; grey bars: unstimulated SDSC). 4 days later a significant difference in metabolic activity was found (B).

Since SDSC are meant to be used in an *in vivo* wound healing model, their growth and stimulation ability in a collagen matrix was analyzed. Therefore, SDSC were cultured in 6 mm pieces of Integra matrix placed on 12-well plates. Scaffolds were incubated at 37°C for improving the attachment of cells to the matrix. Already 4 h post seeding, Phalloidin and DAPI staining visualized the interaction of SDSC with the surrounding matrix. When cultured for 7 days, a homogeneous distribution of viable cells became visible within the matrix (Fig 2A).

Next, the MTT assay revealed that metabolic activity in the cells was evident and present to similar degrees on day 1, 4 and 7 after cell seeding of LPS-stimulated and unstimulated SDSC. On day 11 a difference in metabolic activity was found between the groups (Fig 2B).

### Effect of skin-derived stem cell-conditioned medium on endothelial cells *in vitro*

**Fig 3:**
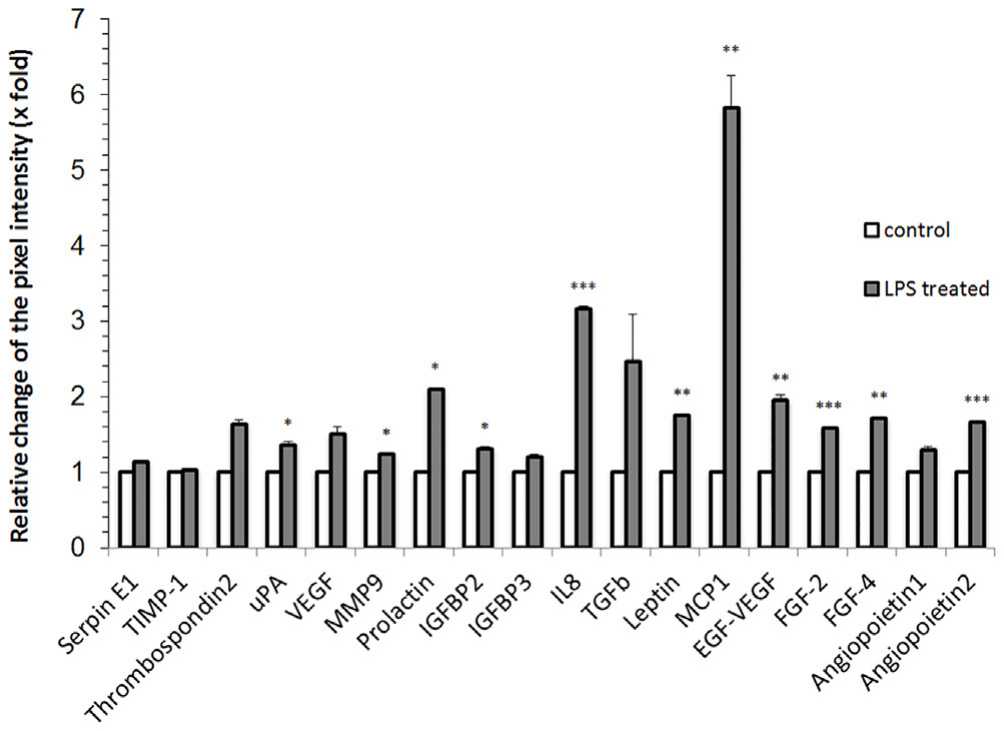
Release of proangiogenic factors by LPS-stimulated SDSC. After 24 hours of cultivation conditioned media were collected (n = 3) and the presence of several bioactive molecules was detected. Proangiogenic factors like IL-8, MCP-1, EGF-VEGF, FGF-2 and Angiopoietin-2 showed a significant increase. Furthermore, uPA, MMP-9, Prolactin, IGFBP-2, Leptin and FGF-4 were significantly elevated. A positive tendency was obvious in Thrombospondin-2, VEGF, TGFb and Angiopoietin-1.

An angiogenesis protein array was performed to analyze the supernatant of SDSC cultured in a collagen matrix 24 h after LPS exposure. Compared to unstimulated SDSC seeded in the scaffold, stimulation of SDSC with LPS led to an enhanced secretion of factors like uPA, MMP-9, Prolactin, IGFBP-2, IL-8, Leptin, MCP-1, EGF-VEGF, FGF-2 and -4 and Angiopoeitin-2. Furthermore, Thrombospondin-2, VEGF, TGFb and Angiopoietin-1 showed a tendency towards an upregulation (Fig 3).

**Fig 4:**
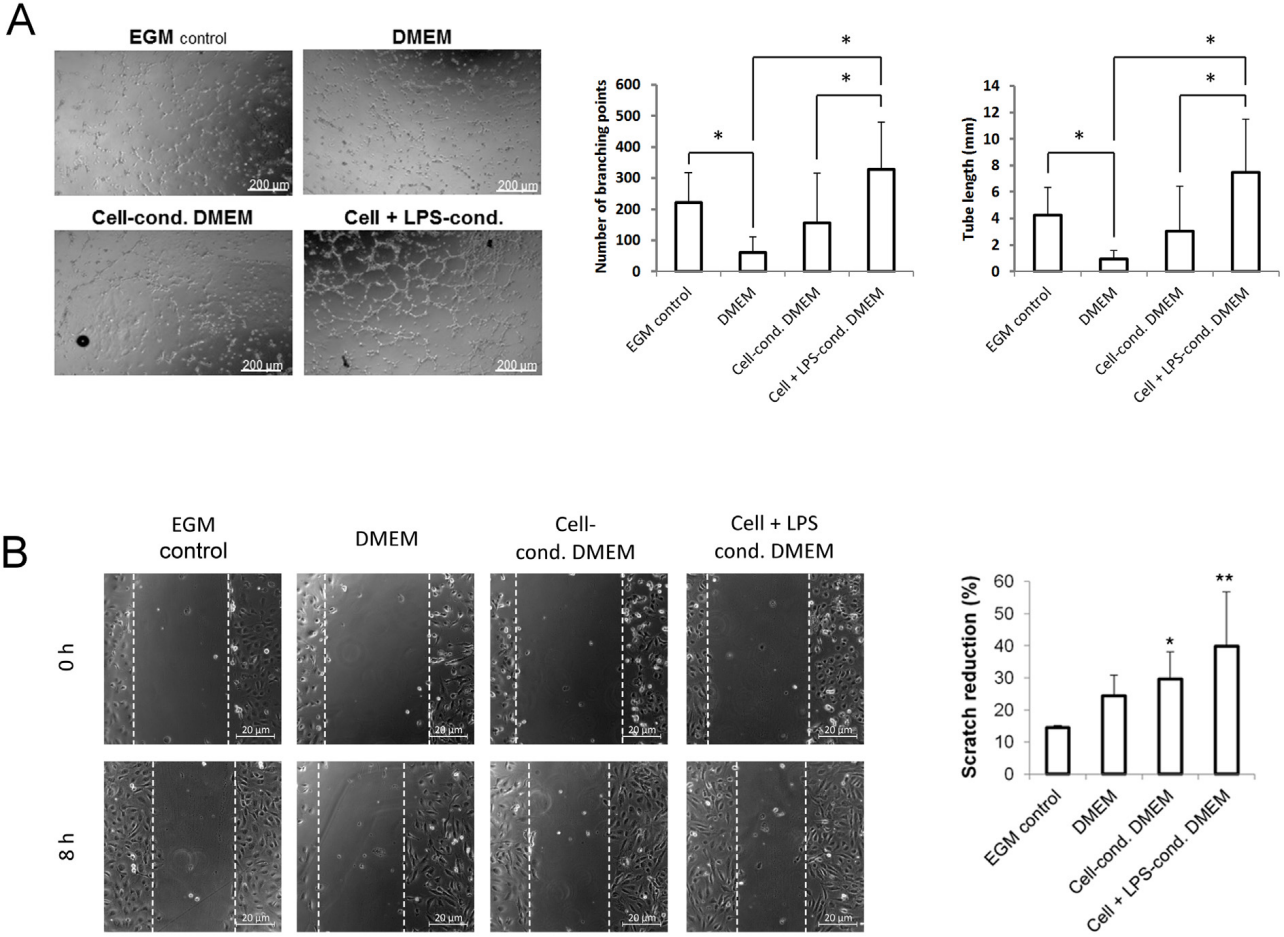
Tube formation and migration of LPS-stimulated SDSC. Human umbilical vein endothelial cells (HUVEC) seeded on Matrigel formed capillary-like structures under the influence of endothelial growth medium (EGM), cell-conditioned DMEM and even more in LPS-treated cell-conditioned DMEM. This effect could not be observed in the DMEM itself (A). In a scratch assay the same media were used showing a significantly higher migration rate when conditioned media were used (B). *p<0.05, **p<0.01, ***p<0.001.

Since these results indicated a proangiogenic effect exerted by stimulated SDSC, a tube formation assay was performed to investigate the respective culture supernatant regarding its potential to possibly support the formation of capillary-like structures. Therefore, human umbilical vein endothelial cells (HUVEC) were seeded onto a matrigel matrix and cultured in different media including SDSC-conditioned 10% FBS-DMEM, LPS-stimulated SDSC-conditioned 10% FBS-DMEM, pure 10% FBS-DMEM and endothelial growth medium (EGM) as positive control. It is known that HUVEC form vessel-like structures in matrigel when cultured in standard EGM. Here, this phenomenon could be observed to occur within 3 h. 10% FBS-DMEM in contrast did not show relevant effects on the morphology of HUVEC. When LPS-stimulated SDSC-conditioned 10% FBS-DMEM was used instead, the cells started to form capillary-like structures in the same manner as in EGM. In addition, the use of LPS-stimulation led to an even more enhanced effect than the use of SDSC-conditioned medium alone (Fig 4A).

Subsequently, the same media as described above were used in an *in vitro* wound closure assay. Here, HUVEC were grown to confluency and a scratch was performed removing cells from a defined area on the culture dish. Over a time frame of 8 h, repopulation of the injured area was then documented. The fastest repopulation was observed using cell-conditioned 10% FBS-DMEM, regardless whether the cells had previously been LPS-stimulated (39.9 ± 16.9% scratch reduction) or not (29.7 ± 8.35% scratch reduction). HUVEC repopulation in conditioned DMEM did significantly differ from the control (Fig 4B). However, no differences were found between EGM and DMEM. The media were also tested in a scratch assay with epithelial cells and fibroblasts. On these cell types the SDSC-conditioned media had no effect on scratch reduction in comparison to standard cultivation medium (data not shown).

### *In vivo* assay

**Fig 5:**
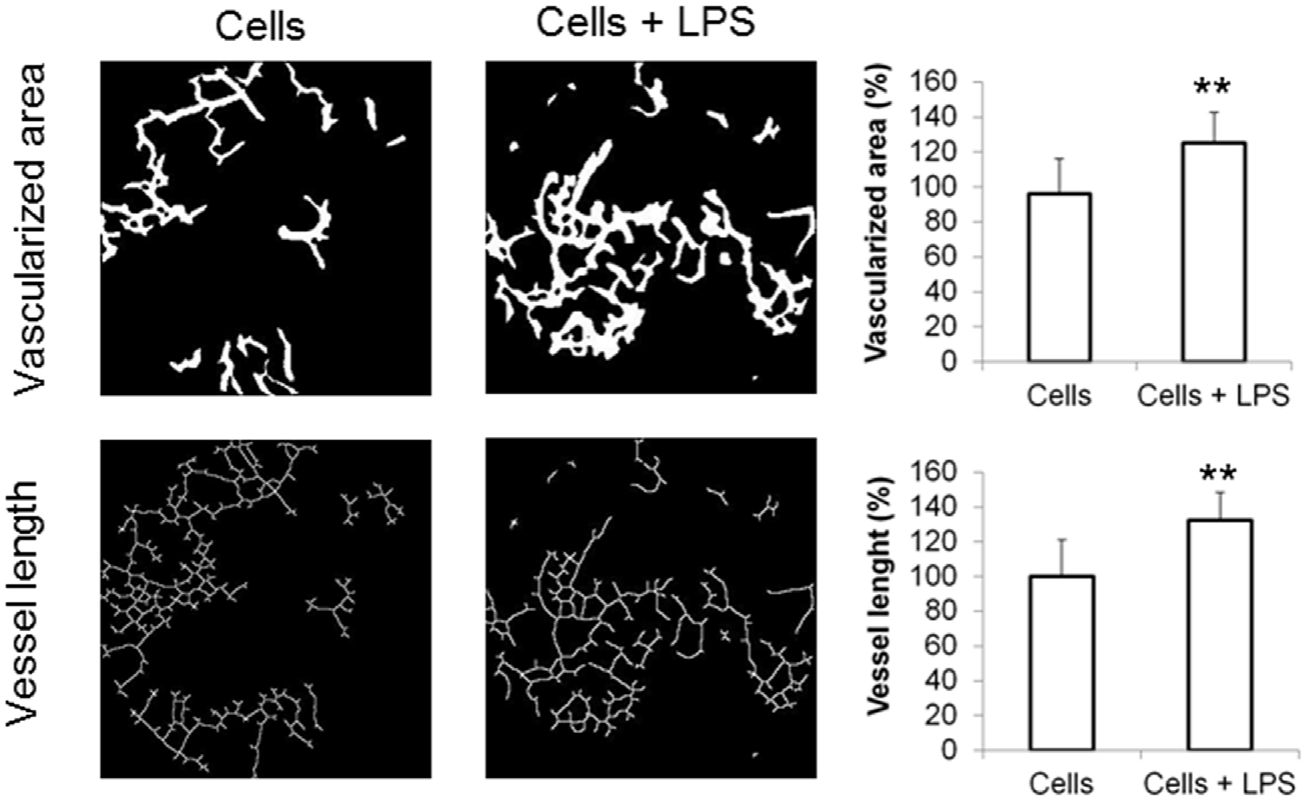
Vascularization of the scaffold *in vivo*. One week after scaffold implantation in a full-thickness wound neovascularization was detected by transillumination and quantification was performed by digital segmentation. Results showed significantly higher vascularized areas and vessel length in the presence of LPS activated SDSC (n = 10) compared to untreated SDSC-seeded scaffolds (n = 10). *p<0.05, **p<0.001.

After confirming the potential of LPS-activated SDSC *in vitro* and their viability when seeded in scaffolds, the cells were engrafted in an *in vivo* full skin defect model. Besides LPS treated SDSC-seeded scaffolds, scaffolds seeded with untreated SDSC have also been analyzed. In order to evaluate the vascularization and wound contraction processes, animals were sacrificed 7 days after engraftment and the whole skin from the back of the animal, including the scaffold, was removed. Tissue transillumination microscopy and digital segmentation were used to quantify vascularization levels. Healthy skin was used as a positive control. The results showed a significant increase in the vessel area when LPS treated SDSC-seeded scaffolds were used (125.1 ± 17.6%), compared to the unstimulated approach (96.0 ± 20.1%). Regarding vessel length, the scaffolds treated with LPS activated SDSC also resulted in significantly higher values (132.4 ± 16.0%) than those seeded with untreated SDSC (100.1 ± 21.0% (n = 10)) (Fig 5). Differences in wound contraction were not found between the groups. Wound contraction in dermal substitutes treated by LPS-stimulated SDSC was 43.7 ± 16.3% and 41.3 ± 12.9%, when SDSC seeded.

## Discussion

In this study, we demonstrated that LPS-stimulated skin-derived stem cells seeded on a collagen scaffold enhance vascularization and subsequently substantiate a regenerative potential in full-thickness wounds.

Besides bone marrow derived cells, mesenchymal stem cells and others, human skin-derived stem cells and sweat gland-derived stem cells exhibit a multipotential differentiation potential and a high proliferation capacity [9–12,22]. For improving vascularization and thereby ensuring oxygen and nutrient reachability for infiltrating cells, scaffolds seeded with mesenchymal and glandular stem cells have successfully been used for skin tissue engineering in the last years [13,14,20]. Collagen matrices and numerous other materials are in use [3]. In the current study we opted for an Integra™ Bilayer Matrix due to its FDA approval and its easy and common clinical usage, e.g. in chronic wounds and burn wounds. Integra consists of a collagen-glycosaminoglycane matrix, containing bovine tendon collagen and chondroitin, covered by a semipermeable silicone membrane, which serves as a temporary barrier to reduce infection rate, saving the matrix from shear stresses in case of dressing changes and controls water vapor loss [1].

Enhancing angiogenesis in the scaffold by achieving permanently high concentrations of growth factors still presupposes the presence of large amounts of stem cells. Transfection of cells with gene vectors has been shown feasible for increasing proangiogenic factor release [4,23], but due to ethical problems alternative methods need to also be evaluated. Therefore, cell stimulation by cytokines after seeding in the scaffold might be less critical and even easier to realize. We could show that cell numbers can effectively be reduced after stimulation with LPS for a comparable enhancement of scaffold vascularization, mediated by an increased proangiogenic factor release. Therefore, we isolated the cells from human full skin biopsies and cultivated them in a 2D and 3D setting. Immunocytochemical staining revealed an expression of TLR-4 on the cell surface, which was confirmed by mRNA analysis, indicating that TLR-4 expression significantly increased 4 hours after LPS-stimulation. However, relative gene expression decreased until 24 hours and remained downregulated. Other methods like flow cytometry were not performed, but might be used in upcoming studies to clarify questions such as how the expression rates change throughout the culturing process or in different subsets of SDSC. Moreover, we did not perform an enrichment of nestin-positive cells, because addition of growth factors that increase the amount of nestin-positivity might have led the cells to differentiate (e.g. towards neural fate). However, we could show that LPS was internalized into the cells after the cells were treated with 10ng/ml of LPS for 1 hour.

The exact mechanisms of LPS uptake still remain unclear. Previously it was shown that LPS uptake in monocytes depends on CD14, while in endothelial cells scavenger receptors pathway is involved [24]. On the other hand, Neal et al. showed that MD2/TLR-4-transfected human endothelial kidney cells were able to internalize E. coli, whereas non-transfected and dominant-negative TLR-4 bearing mutations did not [25]. Furthermore, it was found that endocytosis of LPS-containing liposomes can proceed unassisted by CD14 in clathrin-coated vesicles. Other authors found that the involvement of co-receptors such as CD14 in LPS-effects differs between smooth and rough chemotypes of LPS (sLPS vs. rLPS). Moreover, the concentration of LPS influences the internalization-depending cell response even in absence of CD14 [26]. However, if and how TLR-4 participates in the uptake process of LPS or if it is more involved in LPS signaling remains unanswered and should be evaluated in future studies.

We used 10ng/ml concentration due to previous findings in various kinds of cells. In an aortic endothelium cell line it was shown that a treatment with 10ng/ml increased the intracellular production of heparan sulfate proteoglycan [27], in cultured rat heart myocytes it increased nitric oxide synthase activity [28], it stimulated murine B cell tumor cells to express IgM on the cell surface [29] and it altered the production of a recombinant protein by CHO cells [30]. Furthermore, concentrations of 100ng/ml stimulated the proliferation of T cells and enhanced their production of cytokines [31] and stimulated the release of endothelin in transformed bovine aortic endothelial cultures [32]. On the other hand, Buchman et al. showed that apoptosis can be induced by an exposure of 25ng/ml to porcine aortic endothelial cells [33] and Choi et al. demonstrated that an exposure of 100ng/ml for 24 hours activates caspase-mediated death signaling pathways in endothelial cells [34]. We did not perform apoptosis or cell death analysis in detail, but MTT assay revealed that cells were viable to similar degrees on day 1, 4, and 7 after LPS-stimulation and seeding in the scaffold. At day 11 a significant difference to control group was found, indicating that metabolic activity slightly decreased in LPS-stimulated SDSC. However, most cells were still viable and metabolic activity was still ongoing after LPS-stimulation. To evaluate a dose-response and intracellular pathways such as apoptosis pathways and downstreaming signaling, further studies have to be conducted.

We found that proangiogenic factors like IL-8, MCP-1, EGF-VEGF, FGF-2 and Angiopoeitin-2 significantly increased after LPS-stimulation. Furthermore, uPA, MMP-9, Prolactin, IGFBP-2, Leptin and FGF-4 were elevated. An insignificant positive tendency was obvious in Thrombospondin-2, VEGF, TGFb and Angiopoietin-1.

IL-8 mediates the recruitment of leukocytes, but is also known to play a key role in the building of new vessels [35]. MCP-1 has been shown to be involved in proangiogenic action mediated by stem cells [36]. VEGF levels showed an elevated tendency, but did not reach a significant increase. However, EGF-VEGF was found to be significantly elevated. Furthermore, FGF-2 and Angiopoietin-2 showed a significant upregulation, while Angiopoietin-1 was slightly elevated. Those factors can promote the formation of a stable, long-lasting vasculature network, and might be even more potent than VEGF [37]. While uPA, MMP-9, Leptin and FGF-4 are known to take part in the extracellular matrix degradation and remodeling, VEGF and Thrombospondin-2 influence the vascular network formation. TGFb is known to control proliferation and differentiation of cells, but is also involved in angiogenesis [38]. Additionally, recent data indicates that also Prolactin influences angiogenesis [39]. However, an inflammatory component cannot be excluded.

The ability of HUVEC to form capillary-like structures when cultured with SDSC-conditioned medium after LPS-stimulation was more prominent in comparison with the effect in EGM [40], verifying the proangiogenic effects of the secreted factors. We confirmed these findings in an *in vivo* full-thickness animal model previously described [11,13,14]. After homogeneous distribution of LPS-activated SDSC in a collagen-GAG scaffold engrafted in full-thickness wounds on the back of nude mice, vessel formation was significantly enhanced. These findings are in accordance with recently published data regarding wound healing after direct LPS-stimulation [41], despite an inhibiting component on keratinocyte migration [42]. Even though, it had been thought that proinflammatory factors like TNFα and LPS would exclusively inhibit new vessel growth [43,44], recent data underline their angiogenesis promoting characteristics in adult stem cells [15,45].

However, since we used collagen-glycosaminoglycane scaffolds covered by a silicone membrane, vascularization process could be examined very well, but epithelialization process was not possible to evaluate, because the silicone membrane protects the dermal substitute as an artificial epidermis and concomitantly inhibits the migration of keratinocytes. Wound contraction, on the other hand, should be inhibited by the membrane. Therefore, we examined wound contraction by histological analysis and found that wound contraction was higher than would be expected. However, LPS-stimulation did not alter wound contraction in comparison to control. To investigate the effects on re-epithelialization, keratinocyte survival, graft take and inflammation further studies have to be conducted at different time points using a silicone free dermal substitute.

## Abbreviations

SDSC: Skin-derived Stem Cells
LPS: lipopolysaccharide
EGM: endothelial growth medium
DMEM: Dulbecco’s modified Eagles’s medium
MTT: 3-(4,5-dimethyl-2-thiazolyl)-2,5-diphenyl-2H-tetrazolium bromide
HUVEC: Human Umbilical Vein Endothelial Cells
